# Di-μ-glutarato-κ^4^ *O* ^1^:*O* ^5^-bis­{aqua­[5,6-diphenyl-3-(pyridin-2-yl)-1,2,4-triazine-κ^2^ *N* ^2^,*N* ^3^]copper(II)}

**DOI:** 10.1107/S1600536812022969

**Published:** 2012-05-26

**Authors:** Wei Xu, Lan-Jing-Qian Feng

**Affiliations:** a Center of Applied Solid State Chemistry Research, Ningbo University, Ningbo 315211, People’s Republic of China

## Abstract

In the centrosymmetric dinuclear title complex, [Cu_2_(C_5_H_6_O_4_)_2_(C_20_H_14_N_4_)_2_(H_2_O)_2_], the Cu atom displays a distorted square-pyramidal coordination environment with the basal plane occupied by two 5,6-diphenyl-3-(pyridin-2-yl)-1,2,4-triazine N atoms and two O atoms from different glutarate dianions, while a water mol­ecule is located at the apical position. Of the two water H atoms, one is engaged in an intra­molecular O—H⋯O hydrogen bond, whereas the second is engaged in an inter­molecular O—H⋯O hydrogen bond. The intermolecular hydrogen bonds lead to the formation of a chain along [010].

## Related literature

For the biological activity and applications of triazines, see: Garcia *et al.* (1995 ▶); Mashaly *et al.* (1999 ▶); Soudi *et al.* (2005 ▶).
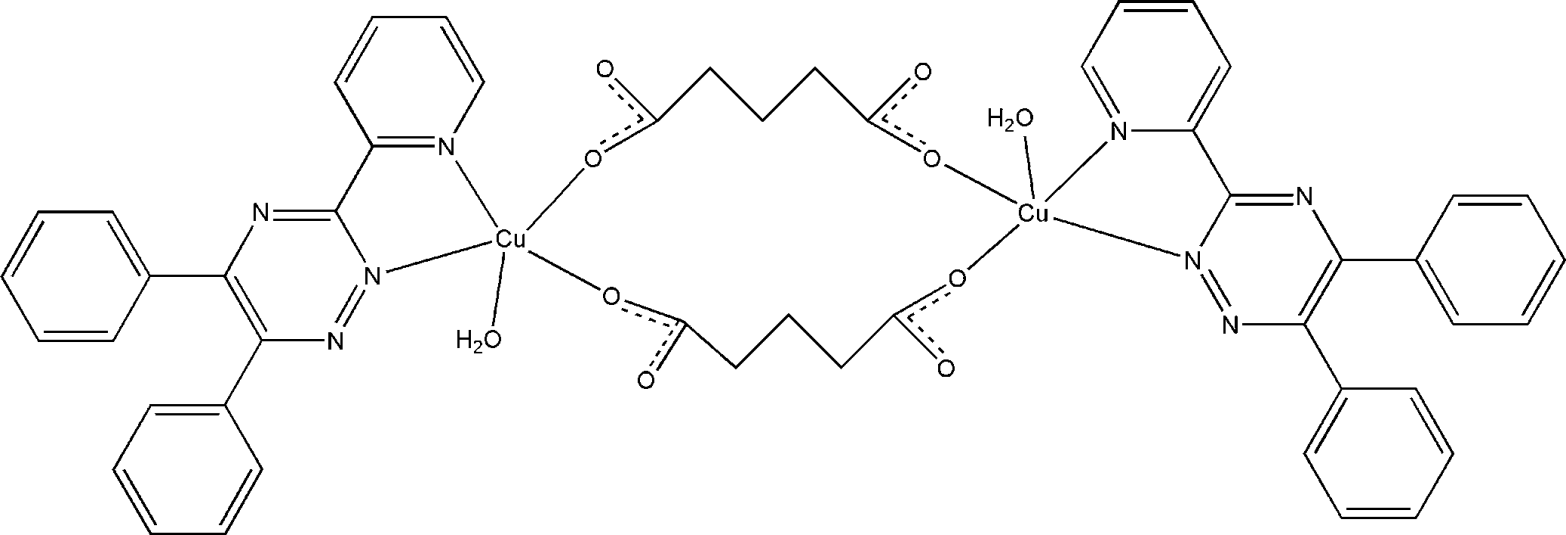


## Experimental

### 

#### Crystal data

[Cu_2_(C_5_H_6_O_4_)_2_(C_20_H_14_N_4_)_2_(H_2_O)_2_]
*M* *_r_* = 1044.01
Triclinic, 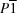

*a* = 9.4297 (19) Å
*b* = 10.429 (2) Å
*c* = 12.471 (3) Å
α = 81.37 (3)°
β = 71.00 (3)°
γ = 79.83 (3)°
*V* = 1135.7 (4) Å^3^
*Z* = 1
Mo *K*α radiation
μ = 1.01 mm^−1^
*T* = 295 K
0.21 × 0.13 × 0.11 mm

#### Data collection

Rigaku R-AXIS RAPID diffractometer
Absorption correction: multi-scan (*ABSCOR*; Higashi, 1995 ▶) *T*_min_ = 0.732, *T*_max_ = 0.854
11269 measured reflections
5148 independent reflections
3478 reflections with *I* > 2σ(*I*)
*R*_int_ = 0.046

#### Refinement

*R*[*F* ^2^ > 2σ(*F* ^2^)] = 0.048
*wR*(*F* ^2^) = 0.107
*S* = 1.03
5148 reflections
324 parameters
3 restraints
H atoms treated by a mixture of independent and constrained refinement
Δρ_max_ = 0.39 e Å^−3^
Δρ_min_ = −0.36 e Å^−3^

### 

Data collection: *RAPID-AUTO* (Rigaku, 1998 ▶); cell refinement: *RAPID-AUTO*; data reduction: *CrystalStructure* (Rigaku/MSC, 2004 ▶); program(s) used to solve structure: *SHELXS97* (Sheldrick, 2008 ▶); program(s) used to refine structure: *SHELXL97* (Sheldrick, 2008 ▶); molecular graphics: *SHELXTL* (Sheldrick, 2008 ▶); software used to prepare material for publication: *SHELXL97*.

## Supplementary Material

Crystal structure: contains datablock(s) global, I. DOI: 10.1107/S1600536812022969/ff2069sup1.cif

Structure factors: contains datablock(s) I. DOI: 10.1107/S1600536812022969/ff2069Isup2.hkl

Additional supplementary materials:  crystallographic information; 3D view; checkCIF report

## Figures and Tables

**Table 1:** Selected bond lengths (Å)

Cu1—O1	1.973 (2)
Cu1—O4^i^	1.917 (2)
Cu1—O5	2.390 (3)
Cu1—N1	2.025 (2)
Cu1—N4	2.033 (2)

Symmetry code: (i) 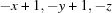.

**Table 2:** Hydrogen-bond geometry (Å, °)

*D*—H⋯*A*	*D*—H	H⋯*A*	*D*⋯*A*	*D*—H⋯*A*
O5—H5*A*⋯O3^i^	0.84	1.94	2.744 (3)	161
O5—H5*B*⋯O1^ii^	0.82	2.25	3.045 (3)	162

Symmetry codes: (i) 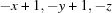; (ii) 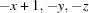.

**Fig. 1.**
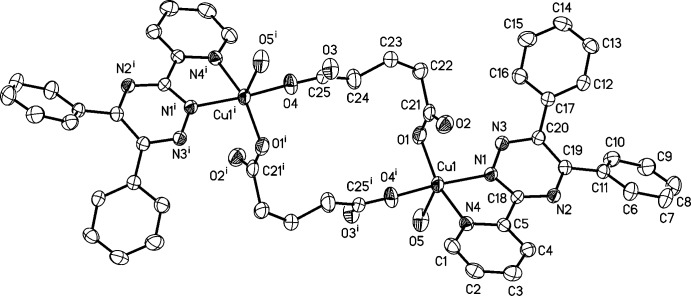
ORTEP view of the title compound (40% thermal ellipsoids) showing the atom-labeling scheme. [Symmetry Code: (i) 1-x, 1-y, -z)]

**Fig. 2.**
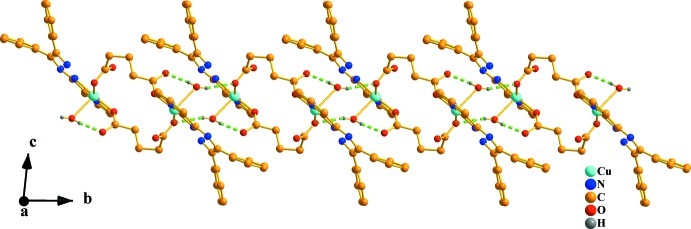
One dimensional chain connected through hydrogen bonds along [010].

## supplementary crystallographic information

### Comment

Numerous compounds containing 1,2,4-triazine moieties are well known in natural materials and show interesting biological, pharmacological and medicinal properties (Garcia *et al.*, 1995). In particularly, the ligand 3-(2-pyridyl)-5,6-diphenyl-1,2,4-triazine (PDPT) exhibits interesting properties such as blood platelet aggregation inhibition, antiviral and anticancer (leukemia and ovarian) and anti-HIV activity (Mashaly *et al.*, 1999; Soudi *et al.*, 2005). The title complex, (I), was recently prepared and its crystal structure is reported here.

The title compound crystal structure is composed of centrosymmetric dinuclear [Cu_2_(H_2_O)_2_(PDPT)_2_(C_5_H_6_O_4_)_2_] complex molecule (Fig. 1). The dinuclear complex molecules are centered at the crystallographic 1*e* positions. Each Cu atom is coordinated to two N atoms of the chelating PDPT ligand and three O atoms of one H_2_O molecule and two bis-monodentate glutarato ligands to form a slightly distorted square-pyramidal coordination with H_2_O molecule located at the apical position (d(Cu-N) = 2.024 (2), 2.033 (2) Å, the basal d(Cu-O) = 1.917 (2), 1.973 (2) Å, the axial d(Cu-O) = 2.390 (3) Å). Through the glutarato ligands, the square-pyramidally coordinated Cu atoms are linked to form a centrosymmetric dinuclear complex. As expected, the Cu atom is shifted toward the apical water O atom by 0.209 (1) Å from the least-squares plane defined by the four equatorial coordinating atoms. The triazine ring adopts a slight twist conformation. The dihedral angle between the two phenyl rings is 61.0 (1)°.

As shown in the Fig. 2, within the crystal structure, the water molecule O5 forms a strong intramolecular hydrogen bond to the uncoordinated carboxyl O3^#1^ (#1 = -x+1, -y+1, -z) with d(O···O) = 2.744 (3) Å and O5-H5A···O3^#1^ = 161°. Moreover, it forms an intermolecular hydrogen bond to the coordinated carboxyl O1^#2^ (#2 = -x+1, -y, -z) atoms (d(O···O) = 3.045 (3) Å and O5-H5B···O1^#2^ = 162°) to connect the dinuclear complexes along the [010] direction.

### Experimental

Addition of 2.0 mL (1.0 M) NaOH to a stirred aqueous of 0.172 g (1.0 mmol) CuCl_2_.2H_2_O in 5.0 mL H_2_O yield a blue precipitate, which was then separated by centrifugation, followed by washing with double-distilled water until no detectable Cl^-^ anions in supernatant. The precipitate was added to a stirred ethanolic aqueous solution of 0.132 g (1.0 mmol) glutaric acid in 20 mL EtOH/H_2_O (v:v = 1: 1). To the resulting suspension was added 0.310 g (1.0 mmol) 3-(2-pyridyl)-5,6-diphenyl-1,2,4- triazine (PDPT). The mixture was further stirred for approximately 15 min and the insoluble solid was filtered off. The filtrate (pH = 6.3) was allowed to stand at room temperature. Slow evaporation for two weeks afforded a small amount of brown crystals (yield 62% based on the initial CuCl_2_.2H_2_O input).

### Refinement

All H atoms bound to C were position geometrically and refined as riding, with C-H = 0.93 Å and *U*_iso_(H) = 1.2*U*_eq_(C). H atoms attached to O were located in difference Fourier maps and placed at fixed positions with *U*_iso_(H) = 1.5*U*_eq_(O).

### Crystal data

**Table:**

[Cu_2_(C_5_H_6_O_4_)_2_(C_20_H_14_N_4_)_2_(H_2_O)_2_]	*Z* = 1
*M**_r_* = 1044.01	*F*(000) = 538
Triclinic, *P*1	*D*_x_ = 1.526 Mg m^−^^3^
Hall symbol: -P 1	Mo *K*α radiation, λ = 0.71073 Å
*a* = 9.4297 (19) Å	Cell parameters from 8151 reflections
*b* = 10.429 (2) Å	θ = 3.3–27.5°
*c* = 12.471 (3) Å	µ = 1.01 mm^−^^1^
α = 81.37 (3)°	*T* = 295 K
β = 71.00 (3)°	Block, brown
γ = 79.83 (3)°	0.21 × 0.13 × 0.11 mm
*V* = 1135.7 (4) Å^3^	

### Data collection

**Table:**

Rigaku R-AXIS RAPID diffractometer	5148 independent reflections
Radiation source: fine-focus sealed tube	3478 reflections with *I* > 2σ(*I*)
Graphite monochromator	*R*_int_ = 0.046
ω scans	θ_max_ = 27.5°, θ_min_ = 3.3°
Absorption correction: multi-scan (*ABSCOR*; Higashi, 1995)	*h* = −12→11
*T*_min_ = 0.732, *T*_max_ = 0.854	*k* = −13→13
11269 measured reflections	*l* = −16→16

### Refinement

**Table:**

Refinement on *F*^2^	Primary atom site location: structure-invariant direct methods
Least-squares matrix: full	Secondary atom site location: difference Fourier map
*R*[*F*^2^ > 2σ(*F*^2^)] = 0.048	Hydrogen site location: inferred from neighbouring sites
*wR*(*F*^2^) = 0.107	H atoms treated by a mixture of independent and constrained refinement
*S* = 1.03	*w* = 1/[σ^2^(*F*_o_^2^) + (0.0413*P*)^2^ + 0.453*P*] where *P* = (*F*_o_^2^ + 2*F*_c_^2^)/3
5148 reflections	(Δ/σ)_max_ < 0.001
324 parameters	Δρ_max_ = 0.39 e Å^−^^3^
3 restraints	Δρ_min_ = −0.36 e Å^−^^3^

### Special details

**Table:**

Geometry. All esds (except the esd in the dihedral angle between two l.s. planes) are estimated using the full covariance matrix. The cell esds are taken into account individually in the estimation of esds in distances, angles and torsion angles; correlations between esds in cell parameters are only used when they are defined by crystal symmetry. An approximate (isotropic) treatment of cell esds is used for estimating esds involving l.s. planes.
Refinement. Refinement of F^2^ against ALL reflections. The weighted R-factor wR and goodness of fit S are based on F^2^, conventional R-factors R are based on F, with F set to zero for negative F^2^. The threshold expression of F^2^ > 2s˘F^2^) is used only for calculating R-factors(gt) etc. and is not relevant to the choice of reflections for refinement. R-factors based on F^2^ are statistically about twice as large as those based on F, and R- factors based on ALL data will be even larger.

### Fractional atomic coordinates and isotropic or equivalent isotropic displacement parameters (Å^2^)

**Table:**

	*x*	*y*	*z*	*U*_iso_*/*U*_eq_	
Cu1	0.26103 (4)	0.21246 (4)	0.03910 (3)	0.03895 (13)	
N1	0.1594 (3)	0.0660 (2)	0.1459 (2)	0.0330 (6)	
N2	−0.0584 (3)	−0.0381 (2)	0.2006 (2)	0.0330 (5)	
N3	0.2040 (3)	−0.0002 (2)	0.2326 (2)	0.0344 (6)	
N4	0.0601 (3)	0.2406 (2)	0.0055 (2)	0.0343 (6)	
C1	0.0116 (4)	0.3395 (3)	−0.0620 (3)	0.0426 (8)	
H1A	0.0752	0.4015	−0.1005	0.051*	
C2	−0.1298 (4)	0.3518 (3)	−0.0763 (3)	0.0455 (8)	
H2A	−0.1617	0.4225	−0.1220	0.055*	
C3	−0.2222 (4)	0.2590 (3)	−0.0225 (3)	0.0454 (8)	
H3A	−0.3162	0.2643	−0.0333	0.054*	
C4	−0.1745 (3)	0.1563 (3)	0.0488 (3)	0.0382 (7)	
H4A	−0.2355	0.0922	0.0866	0.046*	
C5	−0.0341 (3)	0.1529 (3)	0.0614 (2)	0.0310 (6)	
C6	−0.2356 (4)	−0.2275 (3)	0.3326 (3)	0.0444 (8)	
H6A	−0.2892	−0.1488	0.3128	0.053*	
C7	−0.3058 (4)	−0.3385 (3)	0.3674 (3)	0.0526 (9)	
H7A	−0.4070	−0.3343	0.3723	0.063*	
C8	−0.2255 (4)	−0.4556 (3)	0.3950 (3)	0.0569 (10)	
H8A	−0.2729	−0.5305	0.4188	0.068*	
C9	−0.0753 (4)	−0.4623 (3)	0.3875 (3)	0.0515 (9)	
H9A	−0.0211	−0.5422	0.4042	0.062*	
C10	−0.0055 (4)	−0.3516 (3)	0.3553 (3)	0.0418 (7)	
H10A	0.0950	−0.3563	0.3527	0.050*	
C11	−0.0842 (3)	−0.2325 (3)	0.3268 (2)	0.0333 (6)	
C12	0.0555 (3)	−0.1686 (3)	0.5118 (2)	0.0361 (7)	
H12A	−0.0422	−0.1744	0.5128	0.043*	
C13	0.0940 (4)	−0.2000 (3)	0.6113 (3)	0.0417 (7)	
H13A	0.0231	−0.2286	0.6783	0.050*	
C14	0.2369 (4)	−0.1892 (3)	0.6114 (3)	0.0481 (8)	
H14A	0.2627	−0.2096	0.6785	0.058*	
C15	0.3421 (4)	−0.1479 (4)	0.5119 (3)	0.0546 (9)	
H15A	0.4387	−0.1401	0.5122	0.066*	
C16	0.3054 (4)	−0.1181 (3)	0.4117 (3)	0.0454 (8)	
H16A	0.3776	−0.0912	0.3449	0.055*	
C17	0.1608 (3)	−0.1282 (3)	0.4101 (2)	0.0316 (6)	
C18	0.0238 (3)	0.0544 (3)	0.1407 (2)	0.0297 (6)	
C19	−0.0076 (3)	−0.1168 (3)	0.2792 (2)	0.0311 (6)	
C20	0.1183 (3)	−0.0840 (3)	0.3047 (2)	0.0306 (6)	
O1	0.4086 (2)	0.2035 (2)	0.1224 (2)	0.0458 (6)	
O2	0.2028 (3)	0.3259 (2)	0.2159 (2)	0.0537 (6)	
O3	0.4585 (3)	0.7023 (2)	0.1841 (2)	0.0575 (7)	
O4	0.6779 (3)	0.6339 (2)	0.0591 (2)	0.0519 (6)	
O5	0.4157 (3)	0.0709 (2)	−0.1009 (2)	0.0601 (7)	
H5A	0.473 (4)	0.128 (3)	−0.128 (4)	0.090*	
H5B	0.467 (4)	0.000 (2)	−0.093 (4)	0.090*	
C21	0.3332 (4)	0.2717 (3)	0.2053 (3)	0.0422 (8)	
C22	0.4083 (4)	0.2896 (3)	0.2916 (3)	0.0541 (10)	
H22A	0.5054	0.2342	0.2774	0.065*	
H22B	0.3456	0.2632	0.3677	0.065*	
C23	0.4318 (4)	0.4324 (4)	0.2839 (3)	0.0515 (9)	
H23A	0.3353	0.4882	0.2947	0.062*	
H23B	0.4718	0.4435	0.3439	0.062*	
C24	0.5395 (4)	0.4723 (3)	0.1702 (3)	0.0547 (9)	
H24A	0.5087	0.4428	0.1121	0.066*	
H24B	0.6389	0.4250	0.1668	0.066*	
C25	0.5562 (4)	0.6169 (3)	0.1380 (3)	0.0383 (7)	

### Atomic displacement parameters (Å^2^)

**Table:**

	*U*^11^	*U*^22^	*U*^33^	*U*^12^	*U*^13^	*U*^23^
Cu1	0.0377 (2)	0.0393 (2)	0.0407 (2)	−0.01605 (15)	−0.01249 (17)	0.00743 (16)
N1	0.0349 (13)	0.0321 (12)	0.0342 (13)	−0.0096 (10)	−0.0136 (11)	0.0028 (11)
N2	0.0334 (13)	0.0313 (12)	0.0362 (13)	−0.0074 (10)	−0.0131 (11)	−0.0003 (11)
N3	0.0330 (13)	0.0361 (13)	0.0357 (13)	−0.0087 (10)	−0.0127 (11)	0.0014 (11)
N4	0.0376 (14)	0.0326 (13)	0.0325 (12)	−0.0060 (10)	−0.0113 (11)	−0.0001 (11)
C1	0.052 (2)	0.0377 (16)	0.0359 (16)	−0.0070 (14)	−0.0128 (15)	0.0029 (14)
C2	0.051 (2)	0.0442 (18)	0.0381 (17)	0.0055 (15)	−0.0174 (16)	−0.0014 (15)
C3	0.0375 (18)	0.053 (2)	0.0462 (19)	0.0056 (15)	−0.0189 (15)	−0.0073 (16)
C4	0.0360 (17)	0.0423 (17)	0.0371 (16)	−0.0046 (13)	−0.0120 (14)	−0.0061 (14)
C5	0.0332 (15)	0.0309 (14)	0.0284 (14)	−0.0045 (11)	−0.0086 (12)	−0.0028 (12)
C6	0.0412 (18)	0.0360 (16)	0.055 (2)	−0.0099 (13)	−0.0131 (16)	−0.0014 (15)
C7	0.046 (2)	0.0472 (19)	0.067 (2)	−0.0210 (15)	−0.0135 (18)	−0.0054 (18)
C8	0.073 (3)	0.0388 (18)	0.062 (2)	−0.0278 (17)	−0.017 (2)	0.0009 (17)
C9	0.073 (3)	0.0304 (16)	0.058 (2)	−0.0110 (16)	−0.0304 (19)	0.0029 (16)
C10	0.0498 (19)	0.0331 (16)	0.0473 (18)	−0.0065 (13)	−0.0217 (15)	−0.0026 (14)
C11	0.0386 (16)	0.0293 (14)	0.0331 (15)	−0.0109 (12)	−0.0100 (13)	−0.0013 (12)
C12	0.0342 (16)	0.0336 (15)	0.0413 (16)	−0.0096 (12)	−0.0101 (13)	−0.0041 (13)
C13	0.053 (2)	0.0388 (17)	0.0305 (15)	−0.0071 (14)	−0.0076 (14)	−0.0052 (13)
C14	0.051 (2)	0.059 (2)	0.0343 (17)	0.0001 (16)	−0.0182 (16)	−0.0040 (16)
C15	0.0378 (19)	0.080 (3)	0.050 (2)	−0.0037 (17)	−0.0206 (16)	−0.0063 (19)
C16	0.0348 (17)	0.062 (2)	0.0388 (17)	−0.0088 (15)	−0.0104 (14)	−0.0010 (16)
C17	0.0300 (15)	0.0293 (14)	0.0350 (15)	−0.0025 (11)	−0.0108 (12)	−0.0015 (12)
C18	0.0308 (15)	0.0294 (14)	0.0315 (14)	−0.0084 (11)	−0.0103 (12)	−0.0041 (12)
C19	0.0286 (14)	0.0293 (14)	0.0329 (15)	−0.0028 (11)	−0.0065 (12)	−0.0041 (12)
C20	0.0297 (15)	0.0280 (14)	0.0338 (15)	−0.0038 (11)	−0.0100 (12)	−0.0020 (12)
O1	0.0388 (13)	0.0461 (13)	0.0538 (14)	−0.0164 (10)	−0.0141 (11)	0.0035 (11)
O2	0.0437 (14)	0.0599 (15)	0.0596 (15)	−0.0101 (11)	−0.0165 (12)	−0.0073 (12)
O3	0.0535 (15)	0.0505 (14)	0.0608 (16)	−0.0128 (12)	−0.0060 (13)	−0.0016 (13)
O4	0.0501 (14)	0.0419 (13)	0.0554 (14)	−0.0158 (10)	−0.0077 (12)	0.0120 (11)
O5	0.0492 (15)	0.0468 (14)	0.0747 (18)	−0.0111 (11)	−0.0043 (14)	−0.0046 (14)
C21	0.0413 (19)	0.0382 (17)	0.0478 (19)	−0.0208 (14)	−0.0125 (16)	0.0093 (16)
C22	0.067 (2)	0.053 (2)	0.053 (2)	−0.0325 (18)	−0.0311 (19)	0.0170 (18)
C23	0.058 (2)	0.060 (2)	0.0416 (18)	−0.0298 (17)	−0.0155 (17)	0.0042 (17)
C24	0.059 (2)	0.048 (2)	0.052 (2)	−0.0224 (17)	−0.0028 (18)	−0.0015 (17)
C25	0.0393 (18)	0.0438 (18)	0.0355 (16)	−0.0180 (14)	−0.0130 (15)	0.0026 (14)

### Geometric parameters (Å, º)

**Table:**

Cu1—O1	1.973 (2)	C11—C19	1.463 (4)
Cu1—O4^i^	1.917 (2)	C12—C13	1.381 (4)
Cu1—O5	2.390 (3)	C12—C17	1.392 (4)
Cu1—N1	2.025 (2)	C12—H12A	0.9300
Cu1—N4	2.033 (2)	C13—C14	1.373 (5)
N1—C18	1.328 (3)	C13—H13A	0.9300
N1—N3	1.340 (3)	C14—C15	1.377 (5)
N2—C18	1.326 (3)	C14—H14A	0.9300
N2—C19	1.338 (3)	C15—C16	1.380 (4)
N3—C20	1.328 (3)	C15—H15A	0.9300
N4—C5	1.343 (3)	C16—C17	1.392 (4)
N4—C1	1.344 (3)	C16—H16A	0.9300
C1—C2	1.384 (4)	C17—C20	1.483 (4)
C1—H1A	0.9300	C19—C20	1.432 (4)
C2—C3	1.367 (5)	O1—C21	1.282 (4)
C2—H2A	0.9300	O2—C21	1.233 (4)
C3—C4	1.395 (4)	O3—C25	1.221 (4)
C3—H3A	0.9300	O4—C25	1.265 (4)
C4—C5	1.377 (4)	O4—Cu1^i^	1.917 (2)
C4—H4A	0.9300	O5—H5A	0.837 (18)
C5—C18	1.477 (4)	O5—H5B	0.822 (18)
C6—C7	1.378 (4)	C21—C22	1.516 (5)
C6—C11	1.397 (4)	C22—C23	1.528 (5)
C6—H6A	0.9300	C22—H22A	0.9700
C7—C8	1.379 (5)	C22—H22B	0.9700
C7—H7A	0.9300	C23—C24	1.500 (4)
C8—C9	1.378 (5)	C23—H23A	0.9700
C8—H8A	0.9300	C23—H23B	0.9700
C9—C10	1.373 (4)	C24—C25	1.524 (4)
C9—H9A	0.9300	C24—H24A	0.9700
C10—C11	1.391 (4)	C24—H24B	0.9700
C10—H10A	0.9300		
			
O1—Cu1—O5	96.54 (10)	C14—C13—C12	120.0 (3)
O1—Cu1—N1	92.09 (9)	C14—C13—H13A	120.0
O1—Cu1—N4	160.23 (10)	C12—C13—H13A	120.0
O4^i^—Cu1—O1	95.02 (10)	C13—C14—C15	119.9 (3)
O4^i^—Cu1—O5	92.28 (10)	C13—C14—H14A	120.1
O4^i^—Cu1—N1	170.00 (10)	C15—C14—H14A	120.1
O4^i^—Cu1—N4	91.32 (10)	C14—C15—C16	120.6 (3)
N1—Cu1—O5	93.89 (10)	C14—C15—H15A	119.7
N1—Cu1—N4	79.71 (9)	C16—C15—H15A	119.7
N4—Cu1—O5	101.91 (10)	C15—C16—C17	120.3 (3)
C18—N1—N3	118.5 (2)	C15—C16—H16A	119.8
C18—N1—Cu1	115.24 (17)	C17—C16—H16A	119.8
N3—N1—Cu1	124.94 (18)	C12—C17—C16	118.2 (3)
C18—N2—C19	117.5 (2)	C12—C17—C20	121.7 (3)
C20—N3—N1	119.3 (2)	C16—C17—C20	119.8 (3)
C5—N4—C1	118.0 (3)	N2—C18—N1	124.4 (2)
C5—N4—Cu1	115.18 (18)	N2—C18—C5	120.0 (2)
C1—N4—Cu1	126.7 (2)	N1—C18—C5	115.6 (2)
N4—C1—C2	122.1 (3)	N2—C19—C20	117.8 (2)
N4—C1—H1A	118.9	N2—C19—C11	115.4 (3)
C2—C1—H1A	118.9	C20—C19—C11	126.7 (2)
C3—C2—C1	119.2 (3)	N3—C20—C19	119.5 (2)
C3—C2—H2A	120.4	N3—C20—C17	114.0 (2)
C1—C2—H2A	120.4	C19—C20—C17	126.3 (2)
C2—C3—C4	119.5 (3)	C21—O1—Cu1	102.07 (19)
C2—C3—H3A	120.3	C25—O4—Cu1^i^	130.7 (2)
C4—C3—H3A	120.3	Cu1—O5—H5A	89 (3)
C5—C4—C3	117.8 (3)	Cu1—O5—H5B	130 (3)
C5—C4—H4A	121.1	H5A—O5—H5B	109 (3)
C3—C4—H4A	121.1	O2—C21—O1	122.3 (3)
N4—C5—C4	123.2 (3)	O2—C21—C22	118.8 (3)
N4—C5—C18	114.2 (2)	O1—C21—C22	118.9 (3)
C4—C5—C18	122.6 (3)	C21—C22—C23	110.7 (3)
C7—C6—C11	120.4 (3)	C21—C22—H22A	109.5
C7—C6—H6A	119.8	C23—C22—H22A	109.5
C11—C6—H6A	119.8	C21—C22—H22B	109.5
C6—C7—C8	119.8 (3)	C23—C22—H22B	109.5
C6—C7—H7A	120.1	H22A—C22—H22B	108.1
C8—C7—H7A	120.1	C24—C23—C22	110.6 (3)
C9—C8—C7	120.4 (3)	C24—C23—H23A	109.5
C9—C8—H8A	119.8	C22—C23—H23A	109.5
C7—C8—H8A	119.8	C24—C23—H23B	109.5
C10—C9—C8	120.2 (3)	C22—C23—H23B	109.5
C10—C9—H9A	119.9	H23A—C23—H23B	108.1
C8—C9—H9A	119.9	C23—C24—C25	118.4 (3)
C9—C10—C11	120.3 (3)	C23—C24—H24A	107.7
C9—C10—H10A	119.8	C25—C24—H24A	107.7
C11—C10—H10A	119.8	C23—C24—H24B	107.7
C10—C11—C6	118.8 (3)	C25—C24—H24B	107.7
C10—C11—C19	121.3 (3)	H24A—C24—H24B	107.1
C6—C11—C19	119.5 (3)	O3—C25—O4	126.5 (3)
C13—C12—C17	121.0 (3)	O3—C25—C24	121.5 (3)
C13—C12—H12A	119.5	O4—C25—C24	111.9 (3)
C17—C12—H12A	119.5		

Symmetry code: (i) −*x*+1, −*y*+1, −*z*.

### Hydrogen-bond geometry (Å, º)

**Table:**

*D*—H···*A*	*D*—H	H···*A*	*D*···*A*	*D*—H···*A*
O5—H5*A*···O3^i^	0.84	1.94	2.744 (3)	161
O5—H5*B*···O1^ii^	0.82	2.25	3.045 (3)	162

Symmetry codes: (i) −*x*+1, −*y*+1, −*z*; (ii) −*x*+1, −*y*, −*z*.
